# Interactions between the MMP‐3 gene rs591058 polymorphism and occupational risk factors contribute to the increased risk for lumbar disk herniation: A case‐control study

**DOI:** 10.1002/jcla.23273

**Published:** 2020-03-10

**Authors:** Yongjun Luo, Jiaxing Wang, Jie Pei, Yuluo Rong, Wei Liu, Pengyu Tang, Weihua Cai, GuoYong Yin

**Affiliations:** ^1^ Department of Orthopaedics First Affiliated Hospital of Nanjing Medical University Nanjing China; ^2^ Department of Orthopaedics Changzhou TCM Hospital Changzhou China

**Keywords:** lumbar disk herniation, MMP‐3, single nucleotide polymorphism

## Abstract

**Objective:**

Lumbar disk herniation (LDH) is a complex condition based on lumbar disk degeneration (LDD). Previous studies have shown that genetic factors are highly associated with the severity and risk for LDH. This case‐control study was aimed to evaluate the association between the matrix metalloproteinase (MMP)‐3 gene rs591058 C/T polymorphism and LDH risk in a southern Chinese population.

**Methods:**

A total of 231 LDH patients and 312 healthy controls were recruited in this study. Genotyping was analyzed using a standard polymerase chain reaction and restriction fragment length polymorphism (PCR‐RFLP).

**Results:**

It was observed that TT genotype or T allele carriers of the MMP‐3 gene rs591058 C/T polymorphism was more likely associated with an increased risk for LDH. Subgroup analyses showed the following characteristics increased the risk for LDH: female sex; cigarette smoking; and alcohol consumption. Furthermore, individuals with high whole body vibration, bending/twisting, and lifting were associated with an increased risk for LDH.

**Conclusion:**

Taken together, these data indicated that the MMP‐3 gene rs591058 C/T polymorphism was associated with an increased risk for LDH. The MMP‐3 gene rs591058 C/T polymorphism might serve as a clinical indicator and marker for LDH risk in the Chinese population.

## 1 INTRODUCTION

Lumbar disk herniation (LDH) is a common, but intricate spinal degenerative condition affecting approximately 5% of individuals worldwide. LDH leads to nerve root irritation, resulting from mechanical compression or inflammatory mediators, causing low back pain and lower extremity radiating pain, which is also known as sciatica.^1^, ^2^, ^3^ Other genetic and environmental factors, including age, gender, cigarette smoking, occupation, trauma, and obesity, may also play a role in the etiology of LDH.^4^, ^5^ Previous studies have demonstrated that various genetic modifications are involved in LDH progression. The involvement of genetic factors in the etiology LDH has become evident in past decades and likely accounts for up to 70% of an individual's risk. Ala‐Kokko^6^ identified various genes, including collagen IX, vitamin D receptor, and matrix metalloproteinase (MMP)‐3, that are associated with LDH.

The intervertebral disk has a unique outer structure that consists of annulus fibrosus and an elastic inner nucleus pulposus, endowing the intervertebral disk with the capacity to distribute power and resist compression.^7^ One of the major steps of intervertebral disk degeneration is matrix deterioration.^8^ Currently, the mechanism underlying extracellular matrix deterioration, especially the role of gene expression, has become the emphasis of most studies. MMP‐3 is remarkably upregulated and mediates disk degeneration by degrading matrix proteoglycans and collagens during disk degeneration, which indicates the influence on structural modifications of the disk.^9^, ^10^ MMP‐3 gene is located on chromosome 11:102840607. Rs591058 C/T polymorphism is a variant in intron region of MMP‐3 gene. The data from 1000 Genomes show the minor allele frequency of rs591058 C/T is 0.355232. The relationship between MMP‐3 gene rs591058 C/T polymorphism and some disease risk was explored before. Xu et al^11^ found that rs591058 C/T polymorphism had no statistically significant association with frozen shoulder risk. Foster et al^12^ showed that rs591058 C/T polymorphism was associated with the risk of tendon pathology. Raleigh et al^13^ observed that MMP‐3 gene rs591058 C/T polymorphism was associated with Achilles tendinopathy, which possibly interacted with the COL5A1 gene. Recently, an Indian study showed that the MMP‐3 gene rs591058 C/T polymorphism is associated with deceased risk for LDH.^14^ No Chinese studies have addressed this issue up to date. Thus, we conducted this hospital‐based case‐control study to assess the relationship between the MMP‐3 gene rs591058 polymorphism and LDH risk in a Chinese Han population.

## 2 PATIENTS AND METHODS

### 2.1 Subjects

This case‐control study enrolled 231 LDH patients and 312 sex‐ and age‐matched controls from the First Affiliated Hospital of Nanjing Medical University between 2012 and 2018. All MRI images were evaluated by experienced radiologists and orthopedic spine surgeons. Patients with an extruded herniated disk were included in this study. Five potential risk factors for degeneration, including prolonged sitting, bending/twisting, whole body vibration, lifting, and heavy work load, were recorded. Individuals were invited to estimate the number of hours spent per day working affected by each of the five risk factors. The risk factors were classified into the following three levels, which were based on the exposure frequency and years of employment: low; moderate; and high (Table S1). The controls were selected from individuals undergoing health examinations during the same period in the hospital. The individuals with other intervertebral disk diseases were excluded.

Other demographic and risk factor information was extracted from the medical records. This study was approved by the Ethics Committees of the First Affiliated Hospital of Nanjing Medical University and met the standards of the Declaration of Helsinki. Written informed consent was obtained from all subjects.

### 2.2 Blood sampling and genotyping

Blood samples (2 mL) were obtained from the cubital vein of the cases and controls. Genomic DNA was extracted using a TIANamp Blood DNA kit (Tiangen Biotech). The quality and concentration of the extracted DNA were measured at optical density (OD) wavelengths (260 and 280 nm) using NanoDrop (Thermo Scientific). A standard polymerase chain reaction and restriction fragment length polymorphism (PCR‐RFLP) were used for determining the genotypes of the MMP‐3 gene rs591058 C/T polymorphism. Approximately 10% of the selected samples were randomly chosen for genotyping twice to ensure the genotyping accuracy, and the results were 100% concordant.

### 2.3 Statistical analysis

Demographic data from the case and control groups were analyzed using a two‐tailed Student's *t* test (continuous variables) and a chi‐square test (categorical variables), as appropriate. The Hardy‐Weinberg equilibrium (HWE) was also assessed in the control group through a goodness‐of‐fit chi‐square test. Using logistic regression analysis, the odds ratios (ORs) and 95% confidence intervals (CIs) were calculated. Stratified analyses were conducted according to gender, age, alcohol consumption, and cigarette smoking. In addition, the association between the MMP‐3 gene rs591058 C/T polymorphism and factors to which the LDH patients were exposed was explored. All statistical analyses were performed on SPSS 22.0 (SPSS, Inc). The significance level was set at a *P* < .05.

## 3 RESULTS

### 3.1 Characteristics of the study population

The demographic data of cases and controls are summarized in Table 1. The distributions of age, sex, body mass index (BMI), and back injury were not significantly different between the two groups; however, the cigarette smoking rate of cases was higher than the controls. Other clinical data, including prolonged sitting, whole body vibration, heavy workload, bending/twisting, and lifting, are also listed in Table 1. The occupational risk factors for LDH patients are shown in Table S1.

**Table 1:** Patient demographics and risk factors in lumbar disk herniation

Characteristics	Case (N = 231)	Control (N = 312)	*P*
Age	45.7 (29‐78)	47.0 (25‐84)	.161
Sex, n (%)			
Male	146 (63.2%)	179 (57.4%)	.171
Female	85 (36.8%)	133 (42.6%)	
Smoking, n (%)			
YES	127 (55.0%)	140 (44.9%)	**.020**
NO	104 (45.0%)	172 (55.1%)	
Alcohol, n (%)			
YES	117 (50.6%)	146 (46.8%)	.162
NO	114 (49.4%)	166 (53.2%)	
Back injury, n (%)			
YES	74 (32.0%)	38 (12.2%)	**≤.001**
NO	157 (68%)	274 (87.8%)	
Prolonged sitting, n (%)			
Low/Mod	55 (23.8%)		
High	176 (76.2%)		
Whole body vibration, n (%)			
Low/mod	99 (42.9%)		
High	132 (57.1%)		
Heavy workload, n (%)			
Low/mod	72 (31.2%)		
High	159 (68.8%)		
Bending/twisting, n (%)			
Low/mod	62 (26.8%)		
High	169 (73.2%)		
Lifting, n (%)			
Low/mod	85 (36.8%)		
High	146 (63.2%)		

### 3.2 Relationship between the MMP‐3 gene rs591058 C/T polymorphism and the risk for LDH

The HWE test was not significantly different with respect to the genotype frequency of the MMP‐3 gene rs591058 C/T polymorphism in the controls. The TT genotype or TT + CT genotype was associated with an increased risk for LDH (TT vs CC: OR, 1.91; 95% CI, 1.09‐3.37; *P* = .024; TT + CT vs CC: OR, 1.46; 95% CI, 1.03‐2.06; *P* = .034) (Table 2). In addition, the T allele of the rs591058 C/T polymorphism was also associated with an increased risk for LDH (Table 2). Furthermore, the conclusions were still significant after adjusting for age and sex.

**Table 2:** Genotype frequencies of MMP‐3 gene rs591058 polymorphism in cases and controls

Models	Genotype	Case (n, %)	Control (n, %)	OR (95% CI)	*P*‐value	OR (95% CI)*	*P*‐value*
Co‐dominant	CC	88 (38.4%)	148 (47.6%)	1.00 (reference)	‐	‐	‐
Heterozygote	CT	108 (47.2%)	134 (43.1%)	1.36 (0.94‐1.95)	.103	1.38 (0.95‐1.99)	.089
Homozygote	TT	33 (14.4%)	29 (9.3%)	**1.91 (1.09‐3.37)**	**.024**	**2.03 (1.14‐3.63)**	**.017**
Dominant	CC	88 (38.4%)	148 (47.6%)	1.00 (reference)	‐	‐	‐
	TT + CT	141 (61.6%)	163 (52.4%)	**1.46 (1.03‐2.06)**	**.034**	**1.48 (1.04‐2.11)**	**.028**
Recessive	CT + CC	196 (85.6%)	282 (90.7%)	1.00 (reference)	‐	‐	‐
	TT	33 (14.4%)	29 (9.3%)	1.64 (0.96‐2.79)	.069	1.71 (0.99‐2.94)	.054
Allele	C	284 (62.0%)	430 (69.1%)	1.00 (reference)	‐	‐	‐
	T	174 (38.0%)	192 (30.9%)	**1.37 (1.06‐1.77)**	**.015**		

Note

The genotyping was successful in 231 cases and 312 controls for MMP‐3 gene rs591058 polymorphism. Bold values are statistically significant (*P* < .05).

* Adjustment for sex and age.

Stratified analyses were performed according to sex, age, alcohol consumption, and cigarette smoking (Table 3). Increased risk for LDH patients was demonstrated in the females, cigarette smokers, and alcohol drinkers, suggesting that the interactions between these risk factors and the MMP‐3 gene rs591058 C/T polymorphism contributed to the increased risk for LDH.

**Table 3:** Stratified analyses between MMP‐3 gene rs591058 C/T polymorphism and the risk of lumbar disk herniation

Variable	(Case/control)			CT vs CC	TT vs CC	TT vs CC + CT	TT + CT vs CC
	CC	CT	TT				
Sex							
Male	61/84	69/76	14/19	1.25 (0.79‐1.99); 0.345	1.02 (0.47‐2.18); 0.970	0.91 (0.44‐1.88); 0.793	1.20 (0.77‐1.87); 0.412
Female	27/64	39/58	19/10	1.59 (0.87‐2.92); 0.132	**4.50 (1.85‐10.94); 0.001**	**3.51 (1.54‐8.00); 0.003**	**2.02 (1.14‐3.58); 0.016**
Smoking							
Yes	40/67	66/58	20/14	**1.91 (1.13‐3.23); 0.017**	**2.39 (1.09‐5.26); 0.030**	**1.69 (0.81‐3.50); 0.161**	**2.00 (1.21‐3.30); 0.007**
No	48/81	42/76	13/15	0.93 (0.56‐1.57); 0.792	1.46 (0.64‐3.33); 0.366	1.51 (0.69‐3.32); 0.303	1.02 (0.63‐1.66); 0.937
Alcohol							
Yes	35/66	63/68	18/12	**1.75 (1.02‐2.98); 0.041**	**2.83 (1.22‐6.54); 0.015**	**2.05 (0.94‐4.45); 0.069**	**1.91 (1.14‐3.19); 0.014**
No	53/82	45/66	15/17	1.06 (0.63‐1.76); 0.838	1.36 (0.63‐2.97); 0.432	1.33 (0.64‐2.79); 0.447	1.12 (0.69‐1.81); 0.647
Age (y)							
<60	78/128	87/103	19/18	1.39 (0.93‐2.07); 0.110	1.73 (0.86‐3.50); 0.126	1.48 (0.75‐2.90); 0.257	1.44 (0.98‐2.11); 0.064
≥60	10/20	21/31	14/11	1.36 (0.53‐3.48); 0.527	2.55 (0.85‐7.61); 0.095	2.09 (0.85‐5.19); 0.110	1.68 (0.69‐4.03); 0.256

Note

Bold values are statistically significant (*P* < .05).

### 3.3 Correlation between the MMP‐3 gene rs591058 C/T polymorphism and factors to which LDH patients are exposed

Last, we investigated the association between the MMP‐3 gene rs591058 C/T polymorphism and the factors to which the LDH patients are exposed (Table 4). The individuals with high whole body vibration, bending/twisting, and lifting were prone to LDH; however, the prolonged sitting and heavy workload had no effects on the incidence of LDH. To summarize, exposure to high whole body vibration, bending/twisting, and lifting, individuals with the rs591058 C/T polymorphism had an increased risk for developing LDH.

**Table 4:** The associations between MMP‐3 gene rs591058 polymorphism and clinical characteristics of lumbar disk herniation

Characteristics	Genotype distributions			
	CC	CT	TT	CT + TT
Prolonged sitting				
High/Low + Mod	66/22	81/27	27/6	108/33
OR (95%CI); *P*‐value	1.0 (reference)	1.00 (0.52‐1.92); 1.000	1.50 (0.55‐4.12); 0.428	1.09 (0.59‐2.03); 0.783
Whole body vibration				
High/Low + Mod	41/47	64/44	26/7	90/51
OR (95%CI); *P*‐value	1.0 (reference)	1.67 (0.95‐2.94); 0.077	**5.16 (1.94‐13.73); 0.001**	**2.02 (1.18‐3.48); 0.010**
Heavy workload				
High/Low + Mod	58/30	74/34	27/6	101/40
OR (95%CI); *P*‐value	1.0 (reference)	1.13 (0.62‐2.05); 0.698	2.33 (0.87‐6.25); 0.088	1.31 (0.74‐2.32); 0.361
Bending/twisting				
High/Low + Mod	57/31	84/24	28/5	112/29
OR (95%CI); *P*‐value	1.0 (reference)	**1.90 (1.01‐3.58); 0.044**	**3.05 (1.07‐8.68); 0.031**	**2.10 (1.16‐3.82); 0.014**
Lifting				
High/ Low + Mod	47/41	72/36	25/8	97/44
OR (95%CI); *P*‐value	1.0 (reference)	1.75 (0.98‐3.11); 0.059	**2.73 (1.11‐6.70); 0.026**	**1.92 (1.11‐3.33); 0.019**

Note

Bold values are statistically significant (*P* < .05).

## 4 DISCUSSION

In this study, we found that MMP‐3 gene rs591058 C/T polymorphism was related to increased risk for LDH. Subgroup analyses showed increased risk for LDH patients was shown in the female, smokers, and drinkers. In addition, there were synergistic effects between high whole body vibration, bending/twisting, and lifting and the genotypes of rs591058 C/T polymorphism, contributing to increased risk for LDH patients.

MMP‐3 is a member of the MMP family and could mediate disk degeneration via extracellular matrix remodeling, degradation, and connective tissue regeneration.^15^ MMP‐3 is associated with other functions, such as angiogenesis and cell proliferation.^16^, ^17^ Eser et al^18^ observed an association between the increasing level of MMP‐3 gene expression and the radiologic disk herniation grade in a Turkish population, suggesting that MMP‐3 might be a potential clinical indicator for LDH detection and grading.

Recently, MMP‐3 gene polymorphisms were widely evaluated in lumbar disk degeneration (LDD). Takahashi et al^19^ first studied on the effects of MMP‐3 5A/6A polymorphism on the LDD and found that the 5A allele was a risk factor for the acceleration of degenerative changes in the lumbar disk in the elderly. Another study from Egypt 20 with 84 patients and 89 controls showed that the workers with a mutation involving the 5A allele of the MMP‐3 5A/6A polymorphism were more susceptible for LDD. Similar findings were obtained by two Chinese studies,^21^, ^22^ which indicated that individuals with 5A allele carriers exposing to hard labor were more vulnerable to LDH. It is of note that a meta‐analysis showed no association between the MMP‐3 5A/6A polymorphism and interbody disk degeneration susceptibility.^23^ Another SNP (rs632478 polymorphism) was evaluated in an Iranian study,^24^ which suggested the homozygote CC of the MMP‐3 rs632478 polymorphism may lead to an increased risk for IDD compared with the AA genotype.^24^ For this study, we only investigated the MMP‐3 gene rs591058 C/T polymorphism. An Indian study 14 involving 200 LDH patients and 200 healthy controls showed that the TT genotype of the MMP‐3 gene rs591058 C/T polymorphism is a protective factor for LDH in females, which was not consistent with our findings. In this study, we found that the MMP‐3 gene rs591058 C/T polymorphism was associated with an increased risk for LDH. Further analyses observed a significantly increased risk for LDH patients in females, cigarette smokers, and alcohol drinkers. The following factors may account for the conflicting findings of the current study and the Indian study. First, exposure factors were different for these two studies. Second, race differences may also be a potential factor. Third, the clinical heterogeneity of LDH was also different. Fourth, the sample sizes of these studies were important factors that could not be ignored, which may affect the final conclusions. In addition, we investigated the association between the MMP‐3 gene rs591058 C/T polymorphism and the factors affecting LDH patients. The data showed that the individuals with high whole body vibration, bending/twisting, and lifting were prone to LDH.

Cautious consideration for some potential limitations in this present study is in order. First, the correlation between the MMP‐3 gene rs591058 C/T polymorphism and LDH risk could be clearly clarified by a single case‐control study. Second, the sample size was limited, which may underpower the facticity. Third, the lack of population representativeness could not be overlooked because of the ethnic differences. Fourth, we only investigated one SNP of MMP‐3 gene in this study. Furthermore, the occupation, age, and sex discrepancies of the subjects were not taken into consideration, which may cause bias.

Collectively, the MMP‐3 gene rs591058 C/T polymorphism was associated with an increased risk for LDH, suggesting an essential role of this SNP in the progression of LDH; however, the specific mechanism by which the MMP‐3 gene rs591058 C/T polymorphism mediates the occurrence of LDH is unknown, which warrants further study.

## CONFLICT OF INTEREST

The authors declare no conflicts of interest.

## Supporting information

Table S1
