# Supplementary material for: Interactions between the MMP‐3 gene rs591058 polymorphism and occupational risk factors contribute to the increased risk for lumbar disk herniation: A case‐control study
Source: J Clin Lab Anal. 2020 Mar 10;34(7):e23273. doi: 10.1002/jcla.23273 (PMC7370738; doi:10.1002/jcla.23273)
Supplement: Supplementary file 1 — Table S1 [file JCLA-34-e23273-s001.docx]

**Table Two leveled scaled occupational risk factors**

| Risk factor | Level | Description |
| --- | --- | --- |
| Prolonged sitting | Low/mod | ≤4 h/day and ≤1–10 years OR |
| Bending/twisting |  | >4 h/day and ≤ 5 years |
|  | High | ≤4 h/day and >10 years OR |
|  |  | >4 h/day and >5 years |
| Whole body vibration | Low/mod | ≤4 h/day and ≤1–5 years OR |
| Lifting |  | >4 h/day and ≤ 2.5 years |
| Heavy workload | High | ≤4 h/day and >5 years OR  >4 h/day and >2.5 years |
